# Disentangling a Complex of Violet, Endangered Species of *Clavaria* Subsumed Under the Misapplied Name *Clavaria zollingeri* Lév. (Clavariaceae, Fungi)

**DOI:** 10.3390/jof11070482

**Published:** 2025-06-26

**Authors:** Ibai Olariaga, Luis A. Parra, Thomas Læssøe, Juan Manuel Velasco, Ivona Kautmanova, Åsa Kruys, Isabel Salcedo

**Affiliations:** 1 Department Biology and Geology, Physics and Inorganic Chemistry, Rey Juan Carlos University, C/Tulipán s/n, Móstoles, 28933 Madrid, Spain; 2 Aranzadi Society of Sciences, Mycology Section, C/Zorroagagaina 11, 20014 Donostia-San Sebastián, Basque Country, Spain; 3 Independent Researcher, Avda. Miranda do Douro 7, 5º G, Aranda de Duero, 09400 Burgos, Spain; agaricus@telefonica.net; 4 Globe Institute, Department of Biology, University of Copenhagen, Universitetsparken 15, 2100 København Ø, Denmark; thomasl@bio.ku.dk; 5 Independent Researcher, C/Pontevedra 18, 1º C, 37003 Salamanca, Spain; juanmvs@telefonica.net; 6 Slovak National Museum—Natural History Museum, Vajanského Nábrežie 2, P.O. Box 13, 810 06 Bratislava, Slovakia; kautmanova.ivona@gmail.com; 7 Museum of Evolution, Uppsala University, Norbyvägen 16, 752 36 Uppsala, Sweden; asa.kruys@em.uu.se; 8 Department of Plant Biology and Ecology (Botany), University of the Basque Country (UPV/EHU), Apdo 644, 48080 Bilbao, Basque Country, Spain; isabel.salcedo@ehu.eus

**Keywords:** Clavariaceae, *Clavulina*, Zollinger, Java, *Plantae Javanicae*

## Abstract

The name *Clavaria zollingeri* Lév. is currently applied to striking violet species producing branched basidiomata and lacking clamp connections, two typical characteristics of the genus *Clavaria* Pers. Interestingly, as currently interpreted, *C. zollingeri* has been globally assessed as Vulnerable by the IUCN and is red-listed in several European countries. However, the type material of *C. zollingeri*, examined here, possesses clamp connections and should be referred to the genus *Clavulinopsis* Van Overeem. Thus, the name *C. zollingeri* is being misapplied. Based on the taxonomic revision of the specimens, along with morphological and molecular studies of the nrDNA ITS-LSU regions, three species differing in spore characters, basidial size and distribution are recognized. After our nomenclatural revision we conclude that one of the species should be named *Clavaria amethystina* (Holmsk.) Bull., characterized by ellipsoid spores and distribution in the Northern Hemisphere; the second *C. lilacina* Jungh., with subglobose spores and present in Eastern Asia and Oceania; whereas the third, also with subglobose spores and distributed in Europe and North America, is newly described as *C. violaceopulchra*. *Clavaria orientalis* is proposed to be a later synonym of *C. lilacina*. Nine type specimens were examined, the name *C. amethystina* is typified and the combination of *C. zollingeri* in *Clavulinopsis* is proposed.

## 1. Introduction

The name *Clavaria zollingeri* Lév., originally described from Java [1], is applied to one, but in reality several, striking, dichotomously branched and deep violet-colored species (referred to as *C. zollingeri* s. auct. below), the color of which fades to pink with age. *Clavaria zollingeri* s. auct. is distributed in North America [2], South America [3], Asia [4], Oceania [5] and Europe, where it is red-listed in the Czech Republic [6], Denmark [7], Norway [8], Poland [9] and Sweden [10]. More recently, Jordal and Kautmanová [11] assessed *C. zollingeri* s. auct. globally and listed it as Vulnerable. The branched, deeply violet-colored fruitbodies of *C. zollingeri* s. auct. are unique within *Clavaria* L. nom. sanct. Fr. [12], a genus characterized by simple clavarioid fruitbodies, hyaline spores, absence of clamp connections on the context hyphae [13], and basidia of chiastic nuclear division [14,15]. Molecular data also support the placement of *C. zollingeri* s. auct. in *Clavaria* [16,17].

*Clavaria zollingeri* s. auct. was known in Europe before Léveillé introduced the name *C. zollingeri* in 1846 (e.g., [18] (p. 117, *sub Clavaria purpurea* Schaeff.); [19] (p. 110, *sub Ramaria amethystina* Holmsk.); [20] (p. 169, *sub Clavaria amethystina* (Holmsk.) Bull.); [21], (p. 38, *sub C. amethystina*)). Interestingly, earlier names that appear to refer to the same taxon exist—some even based on European material—such as *Clavaria brachycera* Pers. [22] (p. 61), *C. violacea* Vill. [23] (p. 1050), *C. lilacina* Jungh. [1] (p. 216), *C. alcicornis* Zoll. & Moritzi [24] (p. 382) or *C. amethystina* (Holmsk.) Bull. nom. sanct. Fr., but these names are not in current use. Olariaga [25] examined the holotype of *C. zollingeri* and observed clamp connections on the context hyphae, a characteristic suggesting that *C. zollingeri* was not a species of *Clavaria*. Therefore, the name *C. zollingeri* is currently being misapplied and even used for endangered species without clamp connections.

Evidence from various sources suggests that more than one species is subsumed under the name *C. zollingeri* s. auct. Even within Europe, the spore size values provided by different authors show significant variation. While some authors describe the spores as subglobose [25], *sub Clavaria schaefferi* Sacc. [26], others characterize them as ellipsoid [12,27]. Additionally, records assigned to *C. zollingeri* from Australasia and the Americas have been suggested to belong to a different species from that found in Europe [11] as well. Recently, Yan et al. [4] described two new species closely related to *C. zollingeri* based on material from China: *C. orientalis* P. Zhang & Ju. Yan, differing from *C. zollingeri* in its shorter basidia, and *C. tongdaoensis* P. Zhang & Ju. Yan., characterized by a very pale lilac color. Although these authors referred to Corner [12] and Franchi & Marchetti [26], they did not consider or discuss earlier names potentially applicable to the newly proposed taxa. Our analyses of sequences deposited in public databases show a high sequence divergence, supporting also the idea that more than one violet, branched species of *Clavaria* may exist. No global revision including molecular data has been conducted to date.

In this framework, the three main goals of this study are to test species boundaries within the *C. zollingeri* s. auct. complex based on morphological and molecular data, to propose the correct names for the taxa recognized by examining available type specimens, as well as reference specimens and pertinent typifications for each name to stabilize their interpretations.

## 2. Materials and Methods

### 2.1. Herbarium Specimens and Morphological Study

Descriptions were compiled from notes made from fresh material, with further details observed upon dry material. Color codes follow the Royal Horticultural Society [28] for fresh material and Munsell Color Corporation [29] for dry material. Basidiospores were measured in side view from hymenium mountings excluding the apiculus using KOH 5%. Abbreviations referring to basidiospores are the following: L_m_ = mean length, W_m_ = mean width, Q_m_ = L_m_/W_m_; 25 basidiospores were measured per collection. Water and Congo red in ammonia were also used to examine the material. The original literature and nomenclatural types were examined in person. The specimens examined are deposited in ARAN, BCC, BIO, C, BZ, FH, G, H, K, O, PAD, PC, PDD, SALA and UPS herbaria [30]. LAZA refers to the herbarium of the “Sociedad Micológica Salmantina Lazarillo” mycological society. The herbarium specimens examined were filed under the name *Clavaria zollingeri* Lév. unless otherwise stated.

### 2.2. Nomenclature

The Articles and Examples cited in this paper have been extracted from the current International Code for Nomenclature of algae, fungi and plants [31].

### 2.3. DNA Extraction, PCR Amplification, Sequencing and Alignment

DNA was extracted from fresh and dried collections with the DNeasy Plant Mini Kit (QIAGEN, Crawley, West Sussex, UK), following the manufacturer’s protocol. The primer combination ITS5-ITS4 [32] was used to PCR amplify the ITS region, and LR0R [33] and LR5 [33,34] for the LSU region. PCR products were cleaned using ExoSAP-IT^®^ (USB, Cleveland, OH, USA). The PCR amplicons were sequenced in both directions using the same primers. Sequences were edited and assembled using Sequencher v. 4.10 (Gene Codes Corporation Ann Arbor, MI, USA) and have been deposited in GenBank (Table 1). The ITS and LSU sequences were aligned manually in AliView 1.30 [35] using Muscle v5 [36]. Additional sequences were downloaded from the EMBL/GenBank and UNITE databases. Regions that could not be unambiguously aligned were visually detected and removed. Based on Birkebak et al. [37], sequences of *Clavaria fumosa* Pers. (KP257126), *C.* cf. *fumosa* (KP257127) and *C.* cf. *rubicundula* Leathers (HQ877697) were set as outgroup.

### 2.4. Phylogenetic Analyses and OTU Delimitation

The ML analysis was conducted in IQ-TREE [38], starting from a random tree and letting IQ-TREE test the best partitioning scheme under default options. To evaluate branch confidence, 1000 ML bootstrap repetitions were performed using standard bootstrapping. Bootstrap values were considered significant when ≥70% [39]. The Bayesian analysis was carried out in MrBayes v. 3.2.7 [40] via the CIPRES Science Gateway [41]. Two parallel runs of eight Metropolis-coupled Markov chain Monte Carlo (MCMCMC) chains were implemented for 30 M generations, starting from a random tree, and sampling one tree every 100th generation from the posterior distribution. The same partition scheme as in the ML analysis was set, with model parameters unlinked across partitions. Substitution models were sampled across the GTR space during the MCMC simulation [40]. Stationarity was assumed when average standard deviation of split frequencies fell below 0.01. Convergence was further visually diagnosed using Tracer v. 1.7.2. [42]. A burn-in sample of the first 50% of the trees was discarded. To assess branch confidence, a 50% majority rule consensus tree was calculated with the remaining trees using the command SUMT of MrBayes. Bayesian posterior probability (PP) values ≥ 0.95 were considered to be significant.

## 3. Results

### 3.1. Morphological Study

**Figure 1:**
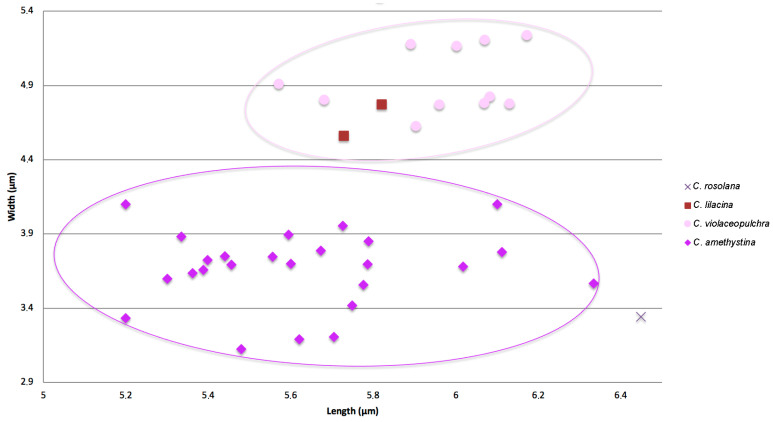
Diagram showing spore measurements from collections within the *Clavaria zollingeri* complex. Spore measurements of *C. rosolana* Petch were obtained from the isotype (K[M]168006).

The L_m_ and W_m_ spore values of thirty-eight sequenced specimens of *C. zollingeri* complex were plotted (Figure 1, Table 1) for comparison, in addition to the spore values of the type material of *C. rosolana*. A group of specimens had ellipsoid spores with a Q_m_ value ranging between 1.27–1.78, whereas another group showed more rounded, subglobose spores, with a Q_m_ range of 1.13–1.28. The spores from the type of *Clavaria rosolana* Petch were the longest (L_m_ = 6.5) and the most ellipsoid ones (Q_m_ = 1.93).

### 3.2. Molecular Study

**Figure 2:**
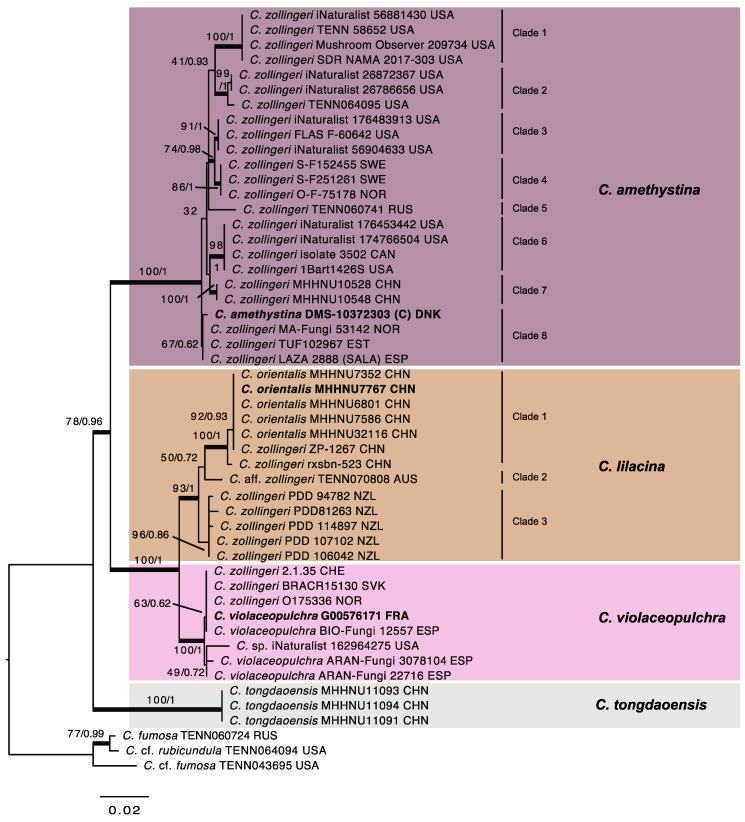
Most Likely Tree from Maximum Likelihood analysis of selected sequences within the *Clavaria zollingeri* s. auct. complex. Maximum Likelihood bootstrap values (ML-Boot)/Bayesian Posterior probabilities (PP) are shown by nodes, ordered as ML-BP/PP. Thickened branches received support in both analyses (ML-BP ≥ 70% and/or PP ≥ 0.95). Voucher specimens and ISO 3166-1 alpha-3 codes [43] for countries are provided after the original identifications of the sequences. Sequences in bold refer to type or reference specimens.

Fifty ITS and nine LSU sequences were generated from fifty specimens (Table 1), from which representative sequences were selected for phylogenetic analyses. The alignment contained 1229 positions and 51 sequences. The ML and Bayesian analyses resulted in consensus trees with highly similar topologies, and congruent, well-supported clades (Figure 2). Specimens assigned to *C. zollingeri* s. auct. formed a large strongly supported clade (ML-BP 78; PP 0.96) basal to the three sequences of *C. tongdaoensis*. The large clade is composed of a subclade attributed here to *C. amethystina* (Holsmk.) Bull. (ML-BP 100; PP 1) and another supported subclade (ML-BP 100; PP 1) that contains two smaller subclades described below as *C. violaceopulchra* (ML-BP 100; PP 1) and *C. lilacina* Jungh. (ML-BP 93; PP 1), respectively. The latter two subclades show long branches, indicating a high sequence divergence between both. The three clades show a sequence divergence that is correlated with their geographic origin. The *C. amethystina* clade contains six supported and one unsupported subclades, as well as a branch composed by a single sequence. Two of the clades contain only North American material, four only European, and two Asian sequences, respectively. The *C. lilacina* clade contains three supported clades composed of material from China, Australia and New Zealand, respectively. The *C. violaceopulchra* clade does not contain any supported clade, but a sequence obtained from North American material is in a long branch. The clade named *C. amethystina* (statistics based on sequenced material alone) is characterized by having very variable, but more ellipsoid (Q_m_ range = [1.27]1.37–1.91) and narrower spores (W_m_ range= 3.1–4.1), whereas the other two, *C. lilacina* (Q_m_ range = 1.22–1.26; W_m_ range = 4.6–4.8) and *C. violaceopulchra* (Q_m_ range = 1.08–1.28; W_m_ range = 3.6–5.2), possess broadly ellipsoid to subglobose spores.

### 3.3. Type Study of Clavaria zollingeri

**Figure 3:**
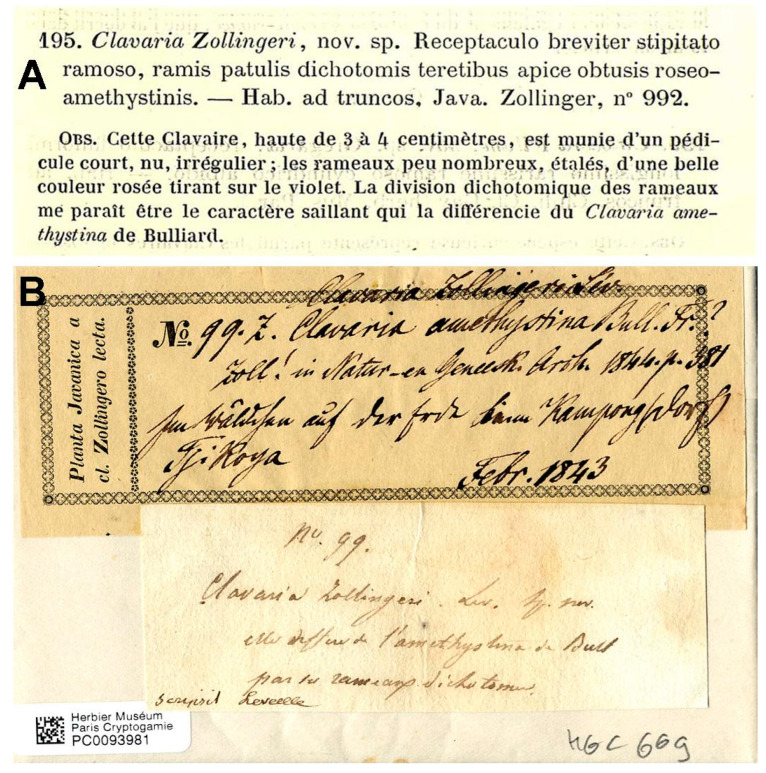
(**A**). Original description of *Clavaria zollingeri* by Léveillé reproduced from Ann. Sci. Nat., Bot., sér. 3, 5: 155. 1846. Source: Biodiversity Heritage Library. (**B**). Label of the holotype collection of *C. zollingeri* Lév. (PC0093981). Photograph: I. Olariaga.

Léveillé [1] (p. 155) described *C. zollingeri* based on a specimen collected by Zollinger in Java, which Zollinger [24] (p. 381) himself had earlier published as *Clavaria amethystina*. Léveillé indicated as collection data of the specimen “Hab. ad truncos, Java. Zollinger, nº 992” (Figure 3A). A type specimen of *C. zollingeri* is housed in PC herbarium (PC0093981), which is kept in the original paper envelope. A printed label of Plantae Javanicae is glued to it, with the following handwritten text: “Clavaria Zollingeri Lév. No 99.z Clavaria amethystina Bull. Fr.? Zoll! In Natur-en Genesch. Arch. 1844. p. 381. Im Wäldchen auf der Erde beim Kampong(dorf). Tjikoya. Febr. 1843”. A second label with a different handwriting is glued on it, reading: “nº 99. Clavaria Zollingeri Lev. sp. nov. elle differe de l’amethystina de Bull par ses rameaux dichotomes. Scripsit Leveille” (Figure 3B). The paper sheet where the specimen is placed bears the notation “ad truncos” on it (seen by I. Olariaga).

The specimen in PC was thus seen by Léveillé. The writing “ad truncos”, and above all, the accompanying Léveillé handwriting demonstrate that it was the single specimen on which Léveillé based his description of *C. zollingeri*. The discrepancy noted by Van Overeem [44] between the collection number given on the label (“99.z”) and the protologue (“992”), is interpreted here as an oversight by Léveillé, since no. 992 in Plantae Javanicae corresponds to the cyperaceous plant “*Fimbristylis albescens* Steud.” [45] (p. 61) and the numbers were unique. Moritzi [46], in his account of the material sent by Zollinger to Europe, and Zollinger himself [45], refer to the *C. zollingeri* material sent to Léveillé as “99.z”. The similarity between “99.z” and “992” may explain the oversight. “Z” refers to specimens containing little material and not duplicated [46] (p. iv). Zollinger [45] (p. 12) himself also marked collection 99.z as “HZ 99” (“Herbarium proprium Zollingeri”), meaning that the specimen was kept in his herbarium [45] (p. ix) and was not distributed. The location of this material is unknown to us (see below). A second discrepancy exists between the habitat information on the label and the protologue. The label reads “Im Wäldchen auf der Erde”, which is translated as “in the small forest on soil”. This conforms to what Zollinger ([24,45], p. 12) and Moritzi [46] (p. 124) cited for the material sent to Léveillé. The inscription “ad truncos” appears to have been added by Léveillé to the paper sheet on which the specimen was kept. The fact that Zollinger and Moritzi consistently cited the specimen sent to Léveillé suggests that “ad truncos” was erroneously written or translated by Léveillé. Regardless of whether this was an error, it is clear that this specimen was seen by Léveillé and that he coined the name *Clavaria zollingeri* based on it.

Another type specimen numbered as Plantae Javanicae 99.z was located at FH within the Patouillard herbarium (FH 00290380). The label bears the same information as the specimen kept at PC, but it is typed. We regard the specimen at PC as the one seen by Léveillé, and hence, the holotype, whereas the specimen at FH is a duplicate, i.e., an isotype. The material kept in Zollinger’s own herbarium could not be found at AMD, B, BO, G, K, L, S or UPS.

**Figure 4:**
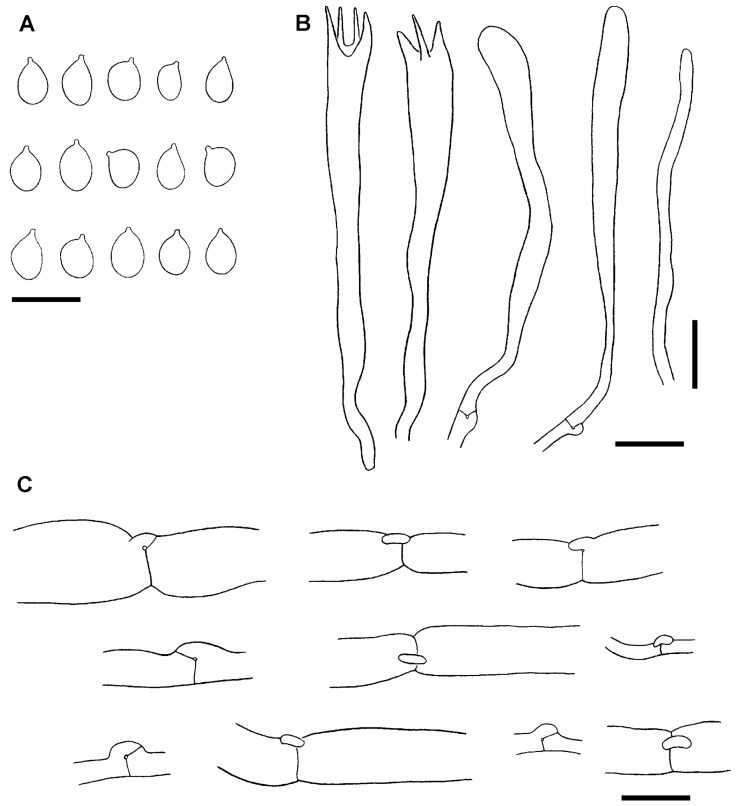
Microscopic structures of the holotype collection of *Clavaria zollingeri* Lév. (PC0093981). (**A**). Basidiospores. (**B**). Basidia and basidioles. (**C**). Hyphae from the context with clamp connections. Scale bars = 10 µm. Author I. Olariaga.

As expected, examination of the holotype and isotype revealed identical morphological characteristics. Both consist of small and fragmented pieces of branches, black, 1–2.7 mm in diam. Microscopically, the structures are collapsed, but relatively well observable when rehydrated in KOH 5%. Basidiospores are broadly ellipsoid, smooth, 5–7 × 3.5–5 µm (L_m_ = 6.1, W_m_ = 4.5, Q_m_ = 1.35; n = 25). Basidia are claviform, long stalked, sometimes sclerified (wall up to 1 µm thick), 44–58 × 5.5–7 µm. The context is formed by a mixture of wide and narrow hyphae, cylindrical to swollen, hyaline, septate, with clamp connections in many septa, 8–12 µm in diam. (Figure 4).

Both type specimens differ from *C. zollingeri* s. auct. in having clamp connections on the context hyphae, a diagnostic character at the generic rank within the Clavariaceae [12,13]. The presence of sclerified basidia, typically found in this family [25,47], suggests that the holotype of *C. zollingeri* belongs to the Clavariaceae lineage within the clavarioid fungi. Due to the presence of clamp connections, hyaline spores and long basidia, we propose below to combine *C. zollingeri* in *Clavulinopsis*.

### 3.4. Taxonomic Treatment

#### 3.4.1. *Clavaria amethystina*

***Clavaria amethystina*** (Holmsk.) Bull., Herb. France 11: Table 496, Figure 2. 1791 [“*amethystea*”] nom. sanct. Fr., Syst. Mycol. 1: 472. 1821. ≡ *Ramaria amethystina* Holmsk., Beata Ruris 1: 110. 1790 [basionym]. ≡ *Clavulina amethystina* (Holmsk.) Donk, Meded. Bot. Mus. Herb. Rijks. Univ. Utrecht 9: 23. 1933. ≡ *Cladaria amethystina* (Holmsk.) Doty, Lloydia 13(1): 14. 1950. Lectotype (designated here): [icon] “Ramaria amethystina” in Holmskjold, Beata ruris 1: t. 11. 1790. MycoBank Typification: MBT 10020236. Reference specimen: Denmark. Midtjylland, Engelsholm, Sønderskov, on earth in broadleaf forest among other clavarioids, *Hygrocybe* spp., *Hodophilus* spp., etc., 21 August 2023, leg. T. Læssøe, DMS-10372303 (C!).

= *Clavaria lavendula* Peck., Bull. New York State Mus. 139: 47. 1910 − Lectotype: USA, Massachusetts, Stow, chestnut grove, 26 July 1909, leg. S. Davis, NYSf1666 (designation achieved by Coker [2] accepting it as the type, according to Art. 7.11).

=? *Clavaria schaefferi* Sacc., Syll. Fung. 6: 693. 1888. [“*schäfferi*”, corrected according to Art. 60.7] [nom. nov. based on *Clavaria lilacina* Fr.]. ≡ *Clavaria lilacina* Fr., Hymenomyc. Eur.: 667. 1874 [nom. illeg. Art. 53, non *Clavaria lilacina* Jungh., Ann. Sci. Nat., Bot., sér. 3, 2: 216. 1844]. ≡ *Clavaria sublilacina* P. Karst., Bidrag Kännedom Finlands Natur Folk 48: 375. 1889. [nom. nov. based on *Clavaria lilacina* Fr.; nom. illeg. Art. 52, the homotypic name *Clavaria schaefferi* with priority ought to have been adopted]. ≡ *Clavaria amethystina* var. *purpurea* Bourdot & Galzin, Hymenomyc. France: 106. 1928. [“1927”]—Lectotype (here designated): Norway, Hordaland, Bergens stift, Etne praestgaard, paa fuktig mark, paa Rottene, 4 August 1864, leg. C. Sommerfelt, UPS F-127181 (!; *in sched. sub Clavaria lilacina*). Mycobank MBT 10026998.

=? *Clavaria rosolana* Petch, Ann. Roy. Bot. Gard. Peradeniya 7(4): 290. 1922—Isotype: Sri Lanka, Waga, on the ground, August 1917, leg. T. Petch 5249 (K(M)168006 (!)).

− *Clavaria purpurea* Schaeff., Fung. Bavar. Palat. Nasc. 4: 117. 1774 *pro parte* (description & plate) *typus* excluded [“*Clvaria*”]

- Description

**Figure 5:**
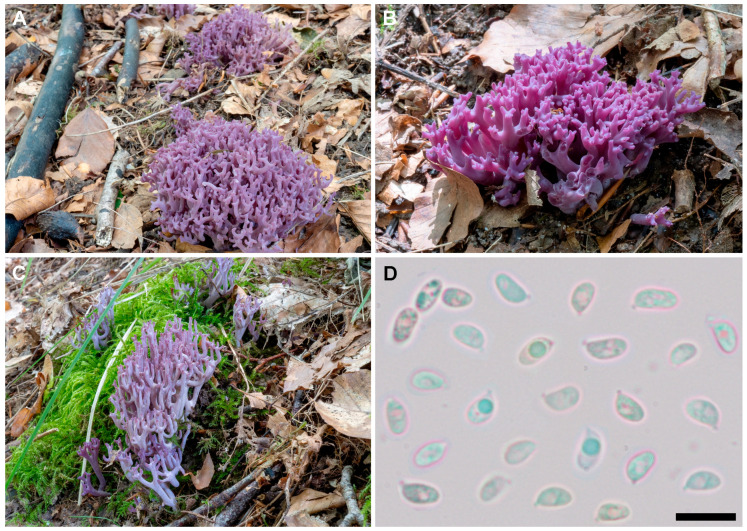
*Clavaria amethystina* (Holmsk.) Bull. (DMS-10372303(C), reference specimen). (**A**–**C**). Basidiomata in situ. (**D**). Basidiospores. Scale bar = 10 µm. Photographs: T. Læssøe (**A**–**C**), I. Olariaga (**D**).

Basidiomata gregarious, 25–60 × 13–25 mm, branched, sometimes with an indistinct stipe. Branching dichotomous to irregularly dichotomous or trichotomous, with U-shaped angles, with a few V-shaped, parallel, branching rank 2–4. Branches cylindrical, solid, bright violet (8A, 8B), fading to pale pink (62D) in age, when dried light ochre (7.5YR 7/2, 8/2) or ochre with a violet hue (5YR 7/2, 8/2). Apices obtuse, sometimes subacute, concolorous or slightly darker. Stipe when present 8–18 × 3–5 mm, cylindrical, bright to paler violet lilac (84A). Whitish mycelium present at the base, seldom forming erect tufts. Context white, taste and odor not recorded. Reaction with iron salts not tested. Basidiospores ellipsoid in side view, a few broadly ellipsoid, thin-walled, smooth, non-amyloid, (4–)5–6.5(–8) × 3–4(–4.5) µm (L_m_ range= 5.2–6.7, W_m_ range = 3.1–3.9, Q_m_ range = (1.27–)1.37–1.91. Basidia clavate, (2–)4-spored, without clamps, 32–42 × 6–8 µm. Context formed by tightly interwoven hyphae, cylindrical to fusiform, closely septate, thin-walled, hyaline to very pale yellow, smooth, without clamps, 4–15 µm broad. Figure 5.

- Distribution and ecology

Widely distributed in the Northern Hemisphere, in temperate areas of continental Europe, confirmed records from Czech Republic, Denmark, Finland, Norway, Slovakia, Sweden, Spain and United Kingdom (following Cotton and Wakefield [48]), North America (Canada, USA) and Asia (China). Records from the Southeast insular Asia to be confirmed. Mainly occurring in non-fertilized pastures in Europe, whereas its prefers habitats are broadleaf and coniferous forests in North America and Asia [4].

- Specimens examined

CZECH REPUBLIC. Czech Republic, Špindlerův Mlýn, Jelení boudy, in mosses and grass in mountain meadow, 27 October 2014, leg. D. Bureš, BRACR21846.

FINLAND. Etelä-Häme Prov., Juupajoki, Hyytiälä Forest Field Station surroundings, in grass, 6 September 2005, leg. S. Jacobsson, BRACR4318. Etelä-Häme Prov., Tammela, Mustiala, 12 August 1866, leg. P.A. Karsten, H6002010, (*in sched. sub C. sublilacina*); duplicate in UPS F-121023, (*in sched. sub C. amethystina*). Varsinais-Suomi, Naantali, Merimasku June 1860, leg. P.A. Karsten, H6081401(!).

FRANCE. Aveyron, Evès, sous des châtaigniers, leg. Galzin, 1 October 1914, Galzin 14845 (UPS).

NORWAY. Agder, Lillesand, Skolehusheia, oak forest with hazel and spruce, 30 September 2023, leg. D. Pettersen & I.L. Fonneland, O-F-260485. Akershus, Nesodden Røer, in mixed deciduous forest, 9 September 2009, leg. V. Kautman, BRACR13263; in broadleaved forest with hazel and oak, 30 August 2008, leg. A. Molia & A.O. Folkestad, O-F-069072. Akershus, Vestby, Gjekstad gard, under *Picea abies*, mossy clearing in the forest, August 2002, leg. P.P. Daniëls, Daniëls 1196 (MA-Fungi 53142). Aust-Agder, Arendal, Rønningheia, in rich deciduous forest, among *Plagiochila asplenoides*, 19 September 2004, leg. T.H. Dahl, O-F-187574. Aust-Agder, Arendal, Tromøy, on bare ground, hazel, oak, lime, 3 August 1998, leg. T.H. Dahl, O-F-090541. Aust-Agder, Grimstad, Tjore, in deciduous forest, hazel, in mosses, 15 September 1999, leg. I.L. Fonneland, O-F-132134. Aust-Agder, Grimstad, Vatnestrand, under hazel, in ground and mosses, 2 September 2000, leg. I.L. Fonneland, O-F-161801. Bomlo, Vestre Vika, sheep grazed seminatural grassland, 6 October 2023, leg. D. Pettersen, BRACR39246. Hedmark, Os, Dalengvollen, in open forest, 21 August 2010, leg. P. Marstad, O-F-244573. Hordaland, Bømlo, Sønstebømakjo, Melhus Lynghei, pasture, 1 October 2011, leg. P. Fadnes & A. Knutsen, O-F-242537. Hordaland, Bergens stift, Etne praestgaard, 22 July 1865, leg. C. Sommerfelt, UPS F-127180 (syntype of *Clavaria schaefferi*, *in sched. sub C. lilacina*). Hordaland, Bømlo, Vestre Vika, in pasture, 25 October 2006, leg. A. Knutsen, O-F-361554. Hordaland, Lindås, Lygra, in pasture, 24 September 2014, leg. J.B. Jordal, O-F-251624. Hordaland, Stord, Kjølsvika, Føyno, shore meadow, 1 October 2011, O-F-242581. Møre og Romsdal, Aure, Husfest, seminatural pasture, 29 September 2020, leg. J.B. Jordal, O-F-312241. Møre og Romsdal, Herøy, Runde, Goksøy, in pasture, 6 October 2016, leg. J.B. Jordal, O-F-254064. Møore og Romsdal, Sande, Sandsøya, Ulandsvika, at the edge of heath, in pasture, 24 September 2009, leg. J.B. Jordal, O-F-291170. Møre og Romsdal, Sunndal, Hagen, in pasture, 8 September 2016, leg. J.B. Jordal, O-F-254066. Møre og Romsdal, Sunndal, Kalvhusvøttu, in pasture, 27 July 1999, leg. J.B.Jordal, O-F-240114. More og Romsdal, Tingvoll, Gyl, in pine forest with scattered deciduous trees, 7 September 2001, leg. G. Gaarder & K. Bang, O-F-176149. Møre og Romsdal, Tingvoll, Liaslettet øst, near road under hazel shrubs, 12 September 2004, leg. G. Gaarder, O-F-360957. Nord Trøndelag, Lierne, Nordli, Kvernvika, in meadow, 12 September 2005, leg. G. Gaarder, O-F-281193. Nord Trøndelag, Steinkjer, Høgmennen, lakeshore, partially outgrown, 7 August 2005, leg. S. Reiso, O-F-285708. Nordland, Hemnes, Solhaug, in pasture, 22 July 2013, leg. G. Gaarder, O-F-2461666. Nordland, Steigen, Hesta sletta ved Laskestad, in rich calcareous pasture, 8 September 2002, leg. G. Gaarder, O-F-223492. Nordland, Vevelstad, Almoselva, in sheep grazed meadow, 1 September 2003, leg. G. Gaarder & T. Hofton, O-F-223396. Oppland, Vestre Toten, Finnstad, in pasture, 17 August 2000, leg. B.H. Larsen & G. Gaarder, O-F-224204. Oslo Fylke, Brannfjell S-V for Svarta, in mosses, in wet shaded forest, 11 July 2002, leg. G. Flatabø, O-F-065543. Rennebu, Hol, Sostuggu, 13 September 2016, leg. S. Vatne, BRACR25781. Rennebu, Rise, 8 September 2016, leg. S. Vatne, BRACR25780. Sogn og Fjordane, Gloppen, Nord for Fella, in cattle pasture, in calcareous soil, in grass, 1 August 2001, leg. G. Gaarder, O-F-176179. Sogn og Fjordane, Selje, Honningsvagen, in pasture, calcareous, 3 October 2000, leg. G. Gaarder & J.B. Jordal, O-F-223561. Sogn og Fjordane, Stryn, Bøasetra, in poor soil, 5 August 2001, leg. G. Gaarder, O-F-176147. Spyssoya, Myra, sheep grazed meadow, 7 October 2023, leg. F. Fuljer, BRACR39145. Steinkjer, Mokk, in grass, 3 September 2009, leg. M. Jeppson & E. Larsson, BRACR13372. Sør-Trøndelag, Oppdal, Gorsetlia, in pasture, 21 August 2008, leg. J.B. Jordal, O-F-287940. Trøndelag, Oppdal, Vinstradalen, in pasture, 21 August 2017, leg. J.B. Jordal, O-F-257161. Vestfold, Hof, Sæteråsen, Lagurtskog, 13 September 2011, leg. T.N. Kristiansen & P. Marstad, O-F-244642. Vestfold, Ramnes, Fossan/Ås, wet ridge, 10 September 1998, leg. K. Geelmuyden, O-F-63441.

SLOVAKIA. Nízke Tatry, Malužiná, Michalovo, in mowed and grazed meadow, 1 September 2010, leg. V. Kautman, BRACR15965. Nízke Tatry, Malužiná village, Michalovo valley, “Nad Gašperíkom”, 22 August 2014, leg. V. Kautman, BRACR36900. Nízke Tatry, Malužiná, Michalovo valley, in pasture, 17 September 2016, leg. I. Kautmanová, BRACR27015. Považský Inovec, Kálnica, Medňanské lúky, pasture, 17 September 2014, leg. J. Herman, BRACR36938. Revúcka vrchovina, Betliar, Straková, 1 July 2013, leg. M. Merva, BRACR24274. Stolicke vrchy, Kokava nad Rimavicou, Háj, in pasture, 10 September 2014, leg. M. Smiková & P. Smik, BRACR28365. Veporské vrchy, Kokava—Háj, pasture, 3 October 2014, leg. V. Kautman, BRACR36929.

SPAIN. Basque Country, Gipuzkoa, Oiartzun, Oieleku, under *Pteridium aquilinum*, on the ground, 10 July 1990, leg. J.M. Lekuona, ARAN-Fungi A3020304. Castilla y León, Salamanca, El Cabaco, La Dehesa, on humus of *Quercus pyrenaica* forest, 26 May 2011, leg. E. Rolo, J.I. Gómez & L.A. Fernández, LAZA 2888 (SALA).

SWEDEN. Gästrikland, Sandviken, Sandvikens kyrka, Åsgatan, i gräsmatta mot kyrkogårdsmuren (in grass), 5 September 1997, leg. O. Lennström, UPS F-013096. Medelpad, Torp sn., Finnsjön, äng (open grassland with scattered trees), 25 August 2009, leg. J.-O. Tedebrand & L. Vessberg, S-F251261). Södermanland, Handen, Getporsvägen 5, lawn in a garden, 19 September 2011, leg. L. Poile, S-F198736. Uppland, Upplands-Väsby, Runby hage, bland gräs och mossa i hagmark (among grass and mosses in grassland), 27 September 1943, leg. L.J. Söderström, UPS F-121017. Värmland, Hagfors kommun, Gustav Adolf församling, Malmbackarna. Naturbetesmark (natural grazed grassland), 17 September 2009, leg. F. Turander, S-F152455. Östergötland, Kvarsebo, 9 September 1951, leg. O. Lundell, UPS F-121011.

- Taxonomic comments

*Clavaria amethystina* appears to be the most common among the violet species of *Clavaria*, and most records from Europe and North America belong to this species. It differs clearly from *C. violaceopulchra* and *C. lilacina* in having more ellipsoid (Q_m_ range = [1.27]1.37–1.91) and narrower (W_m_ range = 3.1–3.9 µm) spores. Macroscopically, basidiomata of *C. amethystina* show less tendency to fade to pink in age. Although the Holmskjold plate selected as the lectotype could represent any of the three violet *Clavaria* species of the complex, the most common species in the Nordic countries—and the only one known from Denmark, also present close to the Holmskjold collection area—is the one with ellipsoid spores, to which we think the epithet *amethystina* should be attached. Accordingly, we propose a reference specimen collected near the Holmskjold collecting area.

In the sanctioning works, under *C. amethystina* Fries [49] made reference to several plates, among which the ones by Schaeffer [50], Holmskjold [19] and Nees von Esenbeck [20] belong to *Clavaria zollingeri* s. auct. in our opinion, while the one by Bulliard [51] clearly shows basidiomata of a species of *Clavulina* J. Schröt, possibly *Cl. coralloides* (L.) J. Schröt parasitized by *Helminthosphaeria clavariarum* (Desm.) Fuckel. Probably due to that, the name *C. amethystina* was interpreted in both ways by later authors: (i) as a *Clavaria* species—as done here (e.g., [2,48,52,53,54]) or (ii) in *Clavulina* J. Schröt. as *Cl. amethystina* (Holmsk.) Donk (e.g., [12,55,56]). The latter interpretation, proposed by Donk [14], was likely based on the Bulliard plate. This interpretation of *C. amethystina* as a species of *Clavulina* is currently accepted (e.g., [26], although the name has been applied to several *Clavulina* species without a consistent taxonomic interpretation [26,57]). Interestingly, many herbarium specimens and publications attributing the name to *Clavulina* (as *Clavulina amethystina*) actually refer to species of *Clavaria* within the *C. zollingeri* s. auct. complex (e.g., [58,59]). Considering this, we follow the interpretation of *C. amethystina* as a *Clavaria* species to provide a stable name for an endangered species, rather than treating it as a *Clavulina* where it would likely become a synonym of *Cl. coralloides* if typified—according to our taxonomic interpretation of the Bulliard plate—or remain a dubious name applied to several species.

Considering that *C. amethystina* is widespread in North America (Figure 2), following previous authors, we treat here *C. lavendula* Peck as a later synonym of *C. amethystina*. Petersen and Olexia [60] examined the type specimen, and the spore measurements provided by them (5.7–6.8 × 2.9–4.0 µm) conform to the material of *C. amethystina* examined by us. The synonymy between *C. lavendula* and *C. amethystina* s. auct. was already proposed ([2,14], synonym under *C. zollingeri*) [12], and considered probable by Petersen and Olexia [60]. Our decision is supported by the fact that *C. amethystina* is widespread in North America and that the *C. amethystina* clade shows a low sequence divergence in North America.

Ellipsoid-spored collections similar to those of *C. amethystina* have been cited from Southeast insular Asia under the names *C. rosolana* Petch [61], *C. zollingeri* [12,44,61,62] and *C. alcicornis* Zoll. & Moritzi [63]. Regarding *C. rosolana*, described from Sri Lanka, the isotype we examined lacked clamp connections, and its spores (5.5–7 × 3–3.5 µm) are also similar to those of *C. amethystina*. We also examined two specimens collected in the Buitenzorg Botanic Garden (25 May 1921, BO 464; 24 May 1921, BO 360) used by Van Overeem [44,62] for his treatments of *C. zollingeri*, which included a beautiful color painting showing ellipsoid spores. Our measurements from BO 464 (4.8–6.5 × 2.5–3.2 µm) were almost identical to the ones provided by him (4.5–6.5 × 2.5–3 µm) and therefore similar to the ones of *C. amethystina*. Additionally, Petersen [63] attributed to *C. zollingeri* s. auct. a specimen labelled as *C. alcicornis* from L (see also Excluded Names) after observing closely septate hyphae with occasional secondary septa and spores measuring 5.9–6.7 × 3.3–4.1 (Q_m_ = 1.69) that conform to *C. amethystina*. From all this evidence, we conclude that *C. amethystina* or a species closely related to it is present in Southeast insular Asia, but further material in better condition and supported by molecular characters would be desirable to confirm this hypothesis. Regrettably, our efforts to loan more recent material from those areas were unfruitful.

- Nomenclatural comments on *Clavaria amethystina* and its synonyms

The nomenclatural background of the names *C. amethystina* and *C. schaefferi* is very intricate with changes in spelling, interpretations of earlier names, invalidly published names, and illegitimate names involved. Therefore, a detailed nomenclatural account of the involved names is deemed necessary.

Some authors (e.g., [2,64]) considered *Coralloides amethystina* Battarra as the basionym of *C. amethystina*. As Donk stated repeatedly [65,66,67,68], with whom we concur, Battarra [69] did not adhere to the binomial system, and according to Art. 23.7(b), even those names that are composed of two words—including *Coralloides amethystina*—must be regarded as not validly published in predominantly polynomial works. Therefore, the basionym of *C. amethystina* is *Ramaria amethystina* Holmsk. [19], as it is the oldest validly published name following a binomial nomenclature system.

*Clavaria amethystina* (Holmsk.) Bull. is a correct combination based on *R. amethystina* Holmsk., even though Bulliard [51] (Table 496, Figure 2) misspelled the original epithet as “amethystea” and misinterpreted the name because his plate shows basidiomata of *Clavulina coralloides* (L.) J. Schröt (=*Clavulina cristata* (Holmsk.) J. Schröt.), parasitized by *Helminthosphaeria clavariarum* (Desm.) Fuckel (see Art. 7.3). Bulliard did not cite any basionym or description in his Herbier de France [51], but he made two references to “Coralloides amethystina BATT. Fung. 22. Tab. I.” and “*Clavaria* purpurea. SCHOEFF. Fung. Tom.II. Table 172” in his Histoire des Champignons de France [70] (p. 200). Those two references were also included by Holmskjold [19] under *Ramaria amethystina*, because both authors considered “Coralloides amethystina” the basionym. Thus, in accordance with Art. 41.4, since Bulliard presumably intended to make a new combination and a potential basionym (*Ramaria amethystina* Holmsk.) applying to the same taxon exists, Bulliard published a valid combination even though he misspelled the original epithet as “amethystea” [51] (p. 496). Fries sanctioned *Clavaria amethystina* in the *Systema Mycologicum* [49] (p. 472, *sub* “*C. amethystina*. Bull. t. 496. f. 2”) and also in the Index of the third volume [71]; (p. 71, *sub* “Clavaria amethystina Bull. I. 472”), referring only to Bulliard. According to Art. F.3.2 last sentence, the spelling used in the sanctioned name is to be maintained and Fries used consistently “amethystina”, the same epithet used in the basionym by Holmskjold.

*Clavaria purpurea* Schaeff. [18] (p. 117) is an illegitimate name (Art. 52) since Schaeffer included the type of *Clavaria palmata* Scop. as synonym, a name that ought to have been adopted. Therefore, nomenclaturally, the name *C. purpurea* becomes a homotypic synonym of *C. palmata*, currently *Thelephora palmata* (Scop.) Fr. nom. sanct., despite the fact that Schaeffer’s plate [50] and description [18] correspond to a *Clavaria* species and not to a *Thelephora* Willd.

Fries was aware that Schaeffer’s plate and description of *C. purpurea* did not match with *T. palmata* and created the name *C. lilacina* [54] (p. 667), based on Schaeffer’s plate and description of *C. purpurea*, excluding the type of *C. palmata* by citing this name under *T. palmata* [54] (p. 634). However, the name *C. lilacina* Fr. is also illegitimate since it is a later homonym of *C. lilacina* Jungh. [1] (p. 216), whereas the replacement names *C. schaefferi* and *C. amethystina* var. *purpurea*, based on *C. lilacina*, are legitimate.

The name *C. sublilacina* P. Karst. is also illegitimate because the homotypic name *C. schaefferi* with priority ought to have been adopted (Art. 52).

*Clavaria schaefferi* is considered here a synonym of *C. amethystina* after examining two syntypes (UPS F-127180, UPS F-127181). Both syntypes belong to *C. amethystina* as treated here. The spores of the syntype in better state (UPS F-127181) measure 5–6 × 3–4 µm and thus conform to our measurements of *C. amethystina*. Thus, this specimen is selected above as lectotype.

#### 3.4.2. *Clavaria lilacina*

***Clavaria lilacina*** Jungh., Ann. Sci. Nat., Bot., sér. 3, 2: 216. Oct. 1844. Lectotype (designated here): Indonesia, Java, ad truncos, without date, PC (!). Mycobank typification: MBT 10025179.

= *Clavaria orientalis* P. Zhang & Jun Yan, Mycokeys 115: 144. 2025. Holotype: China, Hunan Province, Shimen County, Hupingshan Nature Reserve, 11 September 2012, P. Zhang, MHHNU7767.

=? *Clavaria bicolor* Massee, Bull. Misc. Inform. Kew: 154. 1901 [nom. illeg. Art. 53.1] non *Clavaria bicolor* Raf., Med. Repos., ser. [“hexade”] 2, 5: 363. 1808. Holotype: Malaysia, Penang, ad truncos, without date, Ridley, K(M)168004 (!).

- Description

**Figure 6:**
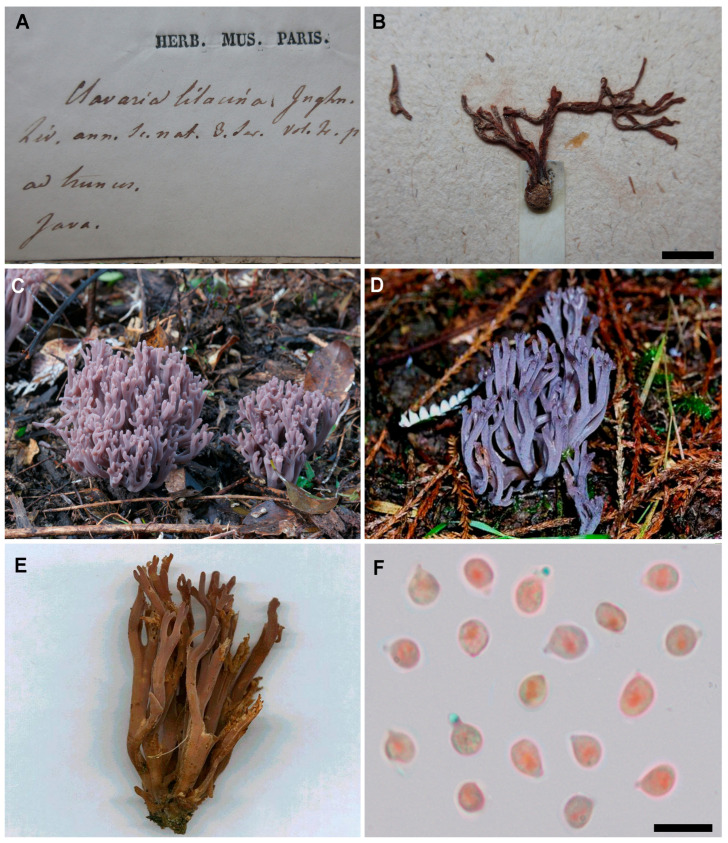
*Clavaria lilacina* Jungh. (**A**) Envelope of the lectotype specimen (PC). (**B**) Single basidioma of the lectotype specimen (PC). (**C**) Basidioma in situ (PDD 94782). (**D**) Basidioma in situ (PDD 81263). (**E**) Aged basidiomata with brown tones (PDD 81263). (**F**) Basidiospores (PDD 81263). Scale bar = 10 µm. Photographs: I. Olariaga (**A**,**B**), Clive Shirley (**C**–**E**), I. Olariaga (**F**).

Basidiomata gregarious, up to 60 × 35 mm, branched, sometimes with a distinct stipe. Branching more or less dichotomous, sometimes trichotomous, with U-shaped angles, parallel or divergent near the apices, branching rank 2–4. Branches cylindrical, sometimes flattened of longitudinally furrowed, solid, bright violet (8A, 8B) to brownish purple (5YR 5/4, 5/3, 7.5YR 5/4, 5/3), fading to pink (9D) in age, when dried light ochre (10YR 8/2, 2.5Y 7/3, 7/4) or very pale lilac (5YR 8/1). Apices obtuse, rounded, concolorous. Stipe when present cylindrical, bright violet (8A, 8B) to brownish purple (5YR 5/4, 5/3, 7.5YR 5/4, 5/3). Whitish mycelium sometimes present at the base. Context white, taste and odor not recorded. Reaction with iron salts not tested. Basidiospores ovoid to subglobose in side view, thin-walled, smooth, non-amyloid, (5–) 5.5–6.5(–7) × (4–) 4.5–5 (–5.5) µm (L_m_ range = 5.7–5.8, W_m_ range = 4.6–4.8, Q_m_ range = 1.22–1.26). Basidia clavate, (2–)4-spored, without clamps, 40–45 × 6–8 µm. Context formed by tightly interwoven hyphae, cylindrical to fusiform, closely septate, thin-walled, hyaline, smooth, without clamps, 7–18 µm wide. Figure 6.

- Distribution and ecology

Eastern Hemisphere, with records confirmed by molecular data from New Zealand, Australia and tropical China, whereas the lectotype specimen is from Indonesia (Java). Records from Malaysia and Salomon Islands [72] are to be referred to this complex and are likely to belong to *C. lilacina* as well. Occurs on soil in forests, rarely (?) also on very decayed wood.

- Specimens examined

NEW ZEALAND. Auckland, Manukau, Murphy’s Bushleg, leg. C. Shirley, 17 June 2004, PDD 81263. Waitakere, Jonkers Road, Forest & Bird, *Leptospermum* & mixed indigenous scrub, leg. C. Shirley, 13 July 2007, CS AK330 (PDD 94782).

- Taxonomic comments

*Clavaria lilacina* is characterized by its branched violet basidiomata, which fade to pink in age, and its subglobose spores. The material examined suggests that *C. lilacina* is morphologically very similar to *C. violaceopulchra*. However, some specimens of *C. lilacina* ([5]; Figure 6E) exhibit a brownish purple color not observed in *C. violaceopulchra*, and the basidia in *C. lilacina* are shorter than in *C. violaceopulchra* (own measurements and [4]). *Clavaria lilacina* appears to be an Eastern hemisphere vicariant of *C. violaceopulchra*. The significant sequence divergence in the ITS region between the *C. lilacina* and *C. violaceopulchra* clades suggests a long-term isolation and supports their separation as well. Sequences within the *C. lilacina* clade show a high sequence divergence correlated with its geographic origin, suggesting that island populations remain isolated. Although further studies including a richer set of material from Australasia and tropical Asia are still needed, our revision revealed that the name *C. zollingeri* should no longer be used to refer to specimens from these areas. *Clavaria tongdaoensis* is a further species of the complex that may be mistaken for *C. lilacina*, from which it differs in a much paler, paler purple to pale purplish pink color [4].

The original specimen of *C. lilacina* kept at PC, examined by us (Figure 6A,B), shows a dichotomously branched basidioma with simple obtuse apices that conforms to the more recent specimens attributed to *C. lilacina* here. The color is reddish brown, as typical in weathered specimens of the species complex treated in this paper. Microscopically, the structures are very collapsed, but no clamp connections were observed by us and the few spores observed measured 6 × 5 µm. Therefore, we propose that this name should be applied to the specimens of Australasia and tropical Asia until a more thorough revision is carried out.

*Clavaria orientalis* P. Zhang & Ju. Yan is considered here a later synonym of *C. lilacina*. Yan et al. [4] described *C. orientalis* primarily based on shorter basidia than noted by Corner [12] and Franchi & Marchetti [26] for *C. zollingeri*, but these authors included also *C. violaceopulchra* in their descriptions, which likely accounts for the observed differences. The protologue of *C. orientalis* did not discuss earlier potentially available names based on Australasian and Asian material, nor did it include sequences originated from material collected in Australia and New Zealand. Although the material from China shows some divergence in the ITS region compared to the material from Australasian collections, we interpret that variation as intraspecific, as morphological differences are absent.

*Clavaria bicolor* Massee is here regarded as a possible synonym of *C. lilacina*, but a full synonymy cannot be proposed here due to the poor condition of the holotype, sent in spirit and now kept in dry state. The basidioma studied was branched above, violaceous grey, lacked clamp connections and had very collapsed subglobose spores (5–5.5 × 3.5–4 µm).

- Nomenclatural comments

The correct authorship of this name should be *C. lilacina* Jungh., and not Jungh ex Lév., following Art. 46.2 (see Ex. 13), since Léveillé is author of the article but not of the whole work—a journal—in which the name was published. *Clavaria lilacina* Jungh. was published in October 1844 according to the footnote found on the bottom left of page 209. From Léveillé’s statement (“des échantillons”) it can be inferred that the original description is based on several specimens deposited at L (“herb. Lugd. Batav.”). The only material labelled as *C. lilacina* at L is a specimen from the Persoon herbarium (L-0713518) which lacks information about its collector and geographic origin and thus cannot be considered original material. This collection is very unlikely to have been made in Java, as all the specimens from the Persoon herbarium referenced by Léveillé’s extensive account in [1] originate from the New World. Conversely, a specimen kept at PC bears a label in Léveillé’s handwriting with the notation “Clavaria lilacina Junghn. Lév. Ann. Sc. nat. 3 ser. vol. 2. p. 216. Ad truncos. Java. Lév.”. This specimen is considered original material and matches our description of *C. lilacina*. Therefore, we designate it as the lectotype.

#### 3.4.3. *Clavaria violaceopulchra*

***Clavaria violaceopulchra*** Olariaga, L.A. Parra, Læssøe, Velasco, Kautman., Kruys & Salcedo, sp. nov. MB 859599.

Holotype: France, Haute-Savoie, Faverges, Englannaz, grassland, 29 June 2006, leg. L. Francini, G00576171 (!).

Etymology: The epithet *violaceopulchra* is a compound coming from the Latin words *violaceus* (violaceous) and *pulchra* (beautiful).

- Description

**Figure 7:**
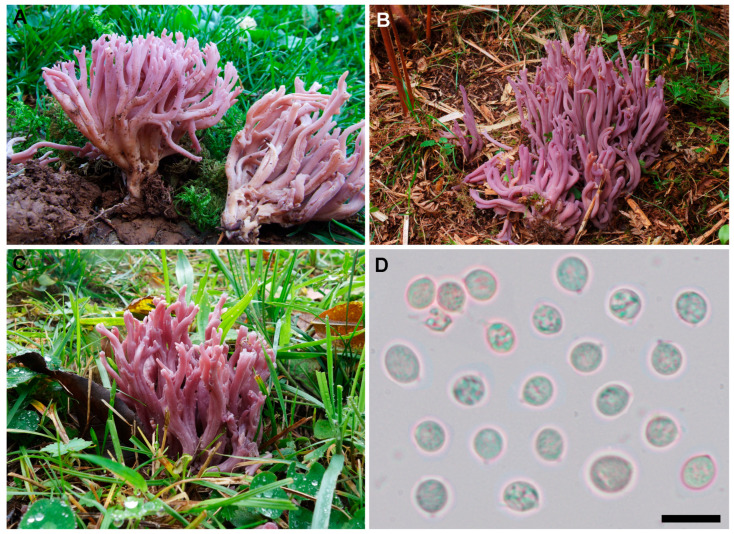
*Clavaria violaceopulchra* sp. nov. (**A**) Basidiomata (G00576171, holotype). (**B**) Basidiomata in situ (BIO-Fungi 12557). (**C**) Basidiomata in situ (ARAN-Fungi 16434). (**D**) Basidiospores (G00576171, holotype). Scale bar = 10 µm. Photographs: L. Francini (**A**), I. Olariaga (**B**–**D**).

Basidiomata gregarious, 42–65 × 14–20 mm, branched, usually without a distinct stipe. Branching irregularly dichotomous, with U-shaped angles, sometimes partly V-shaped, branches parallel or convergent, branching rank 3–4. Branches cylindrical, sometimes flattened, longitudinally furrowed, solid, bright violet (84A), fading to pink (62C, 62D) in age, when dried light ochre (10YR 8/2, 2.5Y 7/3, 7/4) or very pale lilac (5YR 8/1). Apices obtuse, sometimes subacute or truncate, concolorous. Stipe when present 10–15 × 3–5 mm, cylindrical, pinkish lilac (75B, 75C). Whitish mycelium present at the base, seldom forming erect tufts. Context white, taste mild, odor none. No reaction with iron salts. Basidiospores ovoid to subglobose in side view, thin-walled, smooth, non-amyloid, 5–6.5(–7) × 4–6 (–6.5) µm (L_m_ range = 5.4–6.1, W_m_ range = 3.9–5.2, Q_m_ range = 1.08–1.28). Basidia clavate, (2–)4-spored, without clamps, 44–58 × 8–9.5 µm. Context formed by tightly interwoven hyphae, cylindrical to fusiform, closely septate, thin-walled, hyaline to very pale yellow, smooth, without clamps, 4–24 µm wide. Figure 7.

- Distribution and ecology

Mainly temperate areas of central and south Europe (France, Spain, Switzerland), also present in scattered localities of the Nordic countries (Norway) and North America (California), occurring in non-fertilized pastures, periodically cut *Pteridium aquilinum* communities and broadleaf forests, often on acidic ground.

- Distribution and ecology

CZECH REPUBLIC. Rychlebská vrchovina, Jeseník, Smetanovy sady, city park, in grass, 21 October 2017, leg. P. Skopal, BRACR29005.

NORWAY. Aust-Agder, Froland, Syd for Bukkelfjel, Snoøløs, hazel, oak, spruce, on bare ground, leg. 17 December 2001, leg. I. L. Fonneland, O-F-175336.

SLOVAKIA. Biele Karpaty, Veľká Javorina, 28 September 2014, leg. L. Janošík, BRACR23959. Jablunkovské medzihorie, Skalité, sheep grazed meadow, 30 October 2018, leg. M. Zajac, BRACR30724. Javorníky, Veľké Rovné, Solisko, mowed meadow, 2 November 2018, leg. F. Fuljer, BRACR30732. Javorníky, Petrovice, Medvedie, mowed meadow, 3 November 2018, leg. F. Fuljer, BRACR30735. Kysucké Beskydy, Oščadnica-Beskydok, 6 October 2021, leg. V. Kautman, BRACR39564. Kysucké Beskydy, Skalité, 6 October 2021, leg. V. Kautman, BRACR39555. Kysucká vrchovina, Oščadnica, Zadedová, mowed meadow, 15 October 2017, leg. F. Fuljer, BRACR41672. Turzovská vrchovina, Korňa, in meadow, 15 September 2010, leg. L. Mikovčáková, BRACR15130; in shrubs (*Corylus avellana*) on old abandoned pasture, 19 September 2010, leg. L. Mikovčáková, BRACR15909. Veporské vrchy, Kokava-Háj, in pasture, 15 September 2014, leg. P. Pavol, BRACR37053; 10 September 2016, leg. M. Smiková & P. Smik, BRACR82365.

SPAIN. Andalucía, Cádiz, Los Barrios, finca “Murtas”, under *Quercus suber*, *Q. canariensis* and *Olea europaea* var. *sylvestris*, 5 February 2019, leg. M. Romera, ARAN-Fungi 22716. Andalucía, Huelva, Sierra de Aracena, mixed forest with *Pinus* sp., *Quercus suber*, etc., 25 October 2006, leg. S. Silvestre, BIO-Fungi 12538. Basque Country, Gipuzkoa, Berastegi, Artaleku, under *Pteridium aquilinum*, among mosses and gramineous plants, on acid soil, 12 October 2007, leg. J.A. Albizu & I. Olariaga, BIO-Fungi 12557; 7 October 2005, leg. P.M. Pasaban, ARAN-Fungi A3006065. Gipuzkoa, Getaria, San Anton mendia, 12 December 2021, leg. J. Teres, ARAN-Fungi 16434. Gipuzkoa, Zarautz, Santa Barbara, poor-nutrient pasture, 10 December 2010, leg. J. Teres, ARAN-Fungi A3078104. Catalonia, Barcelona, St. Hilari Sacalm, Joanetes, herbes (herbs), 15 November 1998, SCM 3471 (BCC). Navarre, Larraun, Atako bailara, poor grassland among *Pteridium*, 6 September 2002, leg. P.M. Pasaban, J.I. López Amiano, J.M. Lekuona & I. Olariaga, BIO-Fungi 9948; BIO-Fungi 9610.

- Taxonomic comments

*Clavaria violaceopulchra* differs from *C. amethystina*, the other species of this complex present in temperate areas of the Northern Hemisphere, by its subglobose spores. The analyses of the ITS-LSU regions also support its distinctiveness. Both species have a deep violet basidioma color, but the basidiomata of *C. violaceopulchra* are usually larger, less branched and a greater tendency to fade to pale pink in age. According to our measurements, basidia are also longer in *C. violaceopulchra* (44–58 × 8–9.5 µm) than those of *C. amethystina* (32–42 × 6–8 µm) and *Clavaria lilacina* (40–45 × 6–8 µm), and this character could be diagnostic. The latter differs further from *C. violaceopulchra* in sometimes becoming purplish brown in age and an Australiasian distribution.

Some European authors have treated *C. violaceopulchra* under the name *C. amethystina* [48] or as *C. zollingeri* [12,26]. Our nomenclatural revision did not yield any previously published name that can be applied to this taxon, and it is therefore described here as new.

#### 3.4.4. New Combination

***Clavulinopsis zollingeri*** (Lév.) Olariaga comb. nov. MycoBank: MB 853972. ≡ *Clavaria zollingeri* Lév., Ann. Sci. Nat., Bot., sér. 3, 5: 155. 1846 [basionym]. Holotype: Indonesia, Java, Kampong (dorf) Tjikoya, ad truncos, February 1843, leg. Zollinger, *Plantae Javanicae* 99.z (PC0093981 [!]). Isotype: FH 00290380 (!).

#### 3.4.5. Excluded Material

The material studied during this project and found not to belong to *C. zollingeri* s. auct. is provided below.

Specimens examined and excluded. *Clavaria* sp.—SRI LANKA: Henaratgoda, on the ground, 26 July 1916, leg. T. Petch 4840, K(M)168007 [original material of *Clavaria violacea* Petch, nom. illeg.]. *Scytinopogon echinosporus*—INDONESIA: Java, prope Tjikoya, ad terram, leg. Zollinger, Plantae Javanicae 1311, L0836107 [*in sched. sub C. zollingeri*].

#### 3.4.6. Excluded Names

Names that do not belong to *C. amethystina*, *C. violaceopulchra* or *C. lilacina*, but have been referred to these names within the complex.

- ***Clavaria alcicornis*** Zoll. & Moritzi in Zollinger, Natuur-Geneesk. Arch. Ned.-Indië 1: 382. 1844. Lectotype: Indonesia, Java, Tjikoya, March 1843, *Plantae Javanicae* 1125 (FH) (designation achieved by Petersen [73] accepting it as the holotype, according to Arts. 7.11 and 9.10).

An original specimen of *C. alcicornis*, deposited at L, was referred to *C. zollingeri* s. auct. by Petersen [63], as it lacked clamp connections, had typically closely septate context hyphae and ovoid basidiospores (5.8–6.7 × 3.3–4.1 µm) matching *C. lilacina* as interpreted here. However, Petersen [73] had previously examined another original specimen kept at FH. Lectotype designation was achieved by Petersen accepting this specimen at FH as the holotype (Art. 7.11), which is correctable to lectotype (Art. 9.10). Thus, *C. alcicornis* must be interpreted according to its lectotypification. Petersen [73] did not find spores in the lectotype at FH, but he reported 4-spored basidia and clamp connections and attributed it to a species of the genus *Clavulinopsis*. Therefore, the name *C. alcicornis* should be applied to a species of *Clavulinopsis* with pink tones, rather than to the group of *Clavaria* species treated here.

- ***Clavaria amethystina*** subsp. ***coerulescens*** P. Karst., Meddeland. Soc. Fauna Fl. Fenn. 16: 2. 1888. Type specimen: not kept at H (checked in person by I. Olariaga), probably lost.

The brief protologue by Karsten described this taxon as similar in shape and size with *Clavaria flava* Schaeff. nom. sanct. Fr. (currently placed in Ramaria as *R. flava* [Schaeff.] Quél.). The protologue stated that its shape was similar to *Clavaria flava* Schaeff. (≡*Ramaria flava* (Schaeff.) Quél., and thus the name is likely to be referred to *Ramaria* Holmsk.).

- ***Clavaria bizzozeriana*** Sacc., Syll. Fung. 6: 693. 1888. [“Bizzozeriana”][nom. nov. based on *Clavaria tenuissima* Sacc., Michelia 1(4): 436. 1878, nom. illeg. Art. 53, non *Clavaria tenuissima* Lév., Ann. Sci. Nat., Bot., sér. 3, 5: 156. 1846]. ≡ *Ramariopsis bizzozeriana* (Sacc.) Schild, Z. Pilzk. 38: 26. 1972. ≡ *Clavulinopsis bizzozeriana* (Sacc.) Jülich, Int. J. Mycol. Lichenol. 2(1): 120. 1985. Syntypes: Italy, Padova, in uliginosis, October 1878, leg. G. Bizzozero, *Mycotheca Veneta* 1309 (B, BM, BUCM, DBN, E, FH, GE, HBG, K, L, M, PRE, S, SIENA, STR, TLA, TO, W, WRSL).

Saccardo [74] compared *Clavaria bizzozeriana* to *C. lilacina* Fr. in the protologue, noting that *C. bizzozeriana* differed from the latter by having thinner branches, dichotomous branching and smaller spherical spores. Coker [2] examined the syntype deposited at K, describing its spores as 2.5–3.6 µm thick, and considered *C. bizzozeriana* a synonym of *Ramariopsis pulchella* (Boud.) Corner (as *Clavaria pulchella* Boud.), as subsequent authors (e.g., [12,26]) did. The dichotomously branched, slender basidiomata with violet tones and small spherical spores can only represent *R. pulchella* according to our current knowledge, but a modern type examination would be desirable to confirm this view.

- ***Clavaria brachycera*** Pers., Comm. Fung. Clav.: 61. 1797. Type specimen: not kept at L, probably lost.

Persoon described a clavarioid, branched fungus with a stout stipe, branched apices and a violaceous color. He further referred to a plate by Barrelier [75] (Figure 1261) that shows in our opinion a young basidioma of a species of *Ramaria*. The robust stipe and the branching pattern of the apices do not conform to *C. zollingeri* s. auct.

- ***Clavaria nymaniana*** Henn. in Warburg, Monsunia 1: 9. 1900. Type: lost (see below).

*Clavaria nymaniana* Henn. is a further name based on material from Java [76] that has been considered a synonym of *C. zollingeri* [12]. *Clavaria nymaniana* was described in the protologue as having branched violet basidiomata, subglobose to ovoid spores measuring 4.5–5 µm and basidia 25–30 µm long. The basidium size is shorter than the one observed in the *C. zollingeri* s. auct. complex. Important information, such as the presence of clamp connections, is missing in the protologue. Regrettably, no type specimen appears to exist [77], and we are uncertain whether *C. nymaniana* is a later synonym of *C. lilacina* or belongs to another genus in the Clavariaceae (*Clavulinopsis* or *Ramariopsis* Corner).

- ***Clavaria violacea*** Vill., Hist. Pl. Dauphiné 3(2): 1050. 1789. non *Clavaria violacea* Petch, Ann. Roy. Bot. Gard. Peradeniya 7(4): 290. 1922 [nom. illeg. Art. 53.1].

The protologue of *C. violacea* [23] describes a clavarioid species with simple branches and a violaceous color. Villars [23] based the description of *C. violacea* on a handwritten unpublished manuscript by Jullien [78] and included *C. violacea* among the species with unbranched or sparingly branched basidiomata, whereas he included the branches species among the species with divided branches “a tiges divisées”. Jullien [78] himself also characterized *C. violacea* as “Cl. ramis simplicibus acutis violaceis”, denoting a species with simple basidiomata. The term “ramis”, usually translated as “branch” and referring to branched structures, was used by Villars [23] and Jullien [78] in a less common sense—club—to refer to simple basidiomata. Although Fries [79] referred *C. violacea* to *C. amethystina* (p. 287, *sub C. amethystea*), the simple basidiomata described in the protologue of *C. violacea* can hardly refer to the *C. amethystina* s. auct. complex. Rather, we believe that this name could refer to *Alloclavaria purpurea* (O.F. Müll.) Dentinger & D.J. McLaughlin, *Clavaria fumosa* Pers. or even a species of *Clavulina* J. Schröt. Nevertheless, no type specimen of *C. violacea* is preserved in the Villars herbarium (GRM), nor does the protologue refer to any original material or illustration. We therefore regard *C. violacea* as a *nomen ambiguum*.

Villars [23] did not ascribe the name *C. violacea* to Jullien, who had earlier accounted *C. violacea* in a handwritten unpublished manuscript [78] that did not fulfill the requirements for an effective publication (Art. 29.1). Thus, even though Villars acknowledged that Jullien had characterized the species, the name *C. violacea* must be ascribed to Villars [23] alone.

## 4. Discussion

The name *C. zollingeri* has been widely used for several taxa (without clamp connections) not including its type (with clamp connections). The ITS-LSU analyses show that the *C. zollingeri* s. auct. complex contains at least four clades, assigned here to four species. Sequence divergence, particularly in the ITS region, is high in the *C. amethystina* and *C. lilacina* clades, raising the question of whether each encompasses more than one species —with 8 and 3 putative species, respectively. Sequence divergence is strongly correlated with the geographical origin of the specimens, while no consistent morphological differences have been detected within the specimens of each of those three clades. The internal transcribed spacer (ITS) is known to show a high variability across *Clavaria*, even within morphologically rather homogeneous groups, such as the *C. falcata* Pers. and *C. fragilis* Holmsk. clades (see [17]). In our view, the signal of the ITS region in the *C. amethystina* and *C. lilacina* clades should be assessed in the light of analyses of multiple protein-coding markers that will allow for a better interpretation of the signal of the ITS region. In this framework, splitting further *C. amethystina*, *C. lilacina* and *C. violaceopulchra*, considering that no morphological characters support this view, appears premature and unsuitable. Rather, we regard the existing divergence in the ITS region as intraspecific variation, recognizing four species within the *C. zollingeri* s. auct. complex, namely *C. amethystina*, *C. lilacina*, *C. tongdaoensis* and *C. violaceopulchra*.

At least three of those species—*C. amethystina*, *C. lilacina*, and *C. violaceopulchra*— produce branched deep violet basidiomata and have been subsumed under the misapplied name, *C. zollingeri*. Therefore, the name *C. zollingeri* cannot be used for any of those, for which correct names must be adopted. Since *C. zollingeri* is considered globally endangered [11] and the name is often used by non-taxonomists, a first option considered here is to maintain its usage by proposing to conserve *Clavaria zollingeri* with a conserved type that represents one of three species. This choice would, however, require coining a new name for the clamped violet *C. zollingeri* from Java.

As earlier names are available for two of the species treated under the misapplied name *C. zollingeri* s. auct., the choice made by us is to adopt the oldest names available for those two clades, to describe a third clade as new and to combine *C. zollingeri* under *Clavulinopsis*. The name *C. amethystina* as here typified remains attached to the most common, ellipsoid-spored species present in Europe and North America, while *C. violaceopulchra* is described as new for the species so far known from Europe and North America, and *C. lilacina* is proposed to be adopted for the remaining clade present in Australia, New Zealand and China. This decision prevents the name *Clavaria amethystina* (as *Clavulina amethystina* [Holmsk.] Donk) from being used in *Clavulina*, where it is often placed, but it lacks a consistent taxonomic interpretation [57]; see comments under *C. amethystina*).

## Figures and Tables

**Table 1:** Sequenced specimens and their respective original names, UNITE and GenBank accession numbers, mean spore length (L_m_), width (W_m_) and length/width (W_m_). Newly generated sequences are marked in bold.

Updated Identification	Original Identification	ITS	LSU	Voucher	Country	L_m_ × W_m_ (µm)	Q_m_
*C. amethystina*	*C. zollingeri*	KM248911	—	isolate 3502	Canada	—	—
*C. amethystina*	*C. zollingeri*	KP257142	KP257211	TFB11857 (TENN060741)	Russia	—	—
*C. amethystina*	*C. zollingeri*	ON231700	ON228398	MHHNU10548	China	—	—
*C. amethystina*	*C. zollingeri*	ON231699	ON228397	MHHNU10528	China	—	—
*C. amethystina*	*C. zollingeri*	**PV603497**	**—**	BRACR21846	Czech Republic	6.1 × 3.8	1.62
*C. amethystina*	*C. zollingeri*	**PV399839**	**PV400542**	DMS-10372303 (C)	Denmark	5.7 × 3.2	1.78
*C. amethystina*	*C. zollingeri*	UDB022810	—	TUF102967	Estonia	—	—
*C. amethystina*	*C. zollingeri*	**PV603492**	**—**	BRACR4318	Finland	5.5 × 3.7	1.48
*C. amethystina*	*C. zollingeri*	**PV603473**	**—**	BRACR13263	Norway	6.7 × 3.6	1.86
*C. amethystina*	*C. zollingeri*	**PV603474**	**—**	BRACR13372	Norway	5.4 × 3.7	1.47
*C. amethystina*	*C. zollingeri*	**PV603475**	**—**	BRACR25780	Norway	5.4 × 3.8	1.45
*C. amethystina*	*C. zollingeri*	**PV603476**	**—**	BRACR25781	Norway	5.3 × 3.9	1.37
*C. amethystina*	*C. zollingeri*	**PV603477**	**—**	O-F-223396	Norway	5.6 × 3.7	1.51
*C. amethystina*	*C. zollingeri*	**PV603478**	**—**	O-F-223492	Norway	5.2 × 4.1	1.27
*C. amethystina*	*C. zollingeri*	**PV603496**	**—**	O-F-223561	Norway	6.0 × 3.7	1.63
*C. amethystina*	*C. zollingeri*	**PV603479**	**—**	O-F-242537	Norway	5.3 × 3.6	1.47
*C. amethystina*	*C. zollingeri*	**PV603480**	**—**	O-F-244573	Norway	5.6 × 3.9	1.44
*C. amethystina*	*C. zollingeri*	**PV603481**	**—**	O-F-244642	Norway	5.4 × 3.6	1.47
*C. amethystina*	*C. zollingeri*	**PV603482**	**—**	O-F-251624	Norway	6.3 × 3.6	1.78
*C. amethystina*	*C. zollingeri*	**PV603483**	**—**	O-F-254064	Norway	5.6 × 3.7	1.48
*C. amethystina*	*C. zollingeri*	**PV603484**	**—**	O-F-257161	Norway	5.8 × 3.9	1.50
*C. amethystina*	*C. zollingeri*	**PV603498**	**—**	O-F-224204	Norway	—	—
*C. amethystina*	*C. zollingeri*	**PV603495**	**—**	O-F-065543	Norway	—	—
*C. amethystina*	*C. zollingeri*	**PV603494**	**—**	O-F-2461666	Norway	—	—
*C. amethystina*	*C. zollingeri*	**PV603493**	**—**	O-F-312241	Norway	—	—
*C. amethystina*	*C. zollingeri*	**PV603499**	**—**	O-F-63441	Norway	—	—
*C. amethystina*	*C. zollingeri*	**PV603500**	**—**	O-F-161801	Norway	—	—
*C. amethystina*	*C. zollingeri*	**PV603501**	**—**	O-F-287940	Norway	—	—
*C. amethystina*	*C. zollingeri*	**PV603502**	**—**	O-F-69072	Norway	—	—
*C. amethystina*	*C. zollingeri*	UDB035814	—	O-F-75178	Norway	—	—
*C. amethystina*	*C. zollingeri*	**PV404188**	JQ415955	MA-Fungi 53142	Norway	5.6 × 3.2	1.77
*C. amethystina*	*C. zollingeri*	**PV603485**	**—**	BRACR15965	Slovakia	5.8 × 3.7	1.57
*C. amethystina*	*C. zollingeri*	**PV603486**	**—**	BRACR24274	Slovakia	5.7 × 4.0	1.45
*C. amethystina*	*C. zollingeri*	**PV603487**	**—**	BRACR36900	Slovakia	5.4 × 3.7	1.45
*C. amethystina*	*C. zollingeri*	**PV603488**	**—**	BRACR36929	Slovakia	6.1 × 4.1	1.49
*C. amethystina*	*C. zollingeri*	**PV603489**	**—**	BRACR36938	Slovakia	—	—
*C. amethystina*	*C. zollingeri*	**PV603490**	**—**	BRACR27015	Slovakia	—	—
*C. amethystina*	*C. zollingeri*	**PV603491**	**—**	BRACR28365	Slovakia	—	—
*C. amethystina*	*C. zollingeri*	**PV399836**	**PV399846**	LAZA 2888 (SALA)	Spain	5.5 × 3.1	1.76
*C. amethystina*	*C. zollingeri*	**PV399837**	**PV399847**	S-F152455	Sweden	5.8 × 3.6	1.64
*C. amethystina*	*C. zollingeri*	**PV399838**	**PV399848**	S-F251261	Sweden	5.2 × 3.3	1.57
*C. amethystina*	*C. zollingeri*	KP257141	HQ877700	JMB08040912 (TENN064095)	USA	—	—
*C. amethystina*	*C. zollingeri*	ON650097	—	iNaturalist 26872367	USA	—	—
*C. amethystina*	*C. zollingeri*	OR824662	—	iNaturalist 176453442	USA	—	—
*C. amethystina*	*C. zollingeri*	OR800133	—	iNaturalist 174766504	USA	—	—
*C. amethystina*	*C. zollingeri*	ON059229	—	iNaturalist 56904633	USA	—	—
*C. amethystina*	*C. zollingeri*	MH016820	—	FLAS-F-60642	USA	—	—
*C. amethystina*	*C. zollingeri*	HQ021907	—	1Bart1426S	USA	—	—
*C. amethystina*	*C. zollingeri*	OR987432	—	iNaturalist 176483913	USA	—	—
*C. amethystina*	*C. zollingeri*	ON650096	—	iNaturalist 26786656	USA	—	—
*C. amethystina*	*C. zollingeri*	AY854071	AY639882	TENN 58652	USA	—	—
*C. amethystina*	*C. zollingeri*	ON650095	—	Mushroom Observer 209734	USA	—	—
*C. amethystina*	*C. zollingeri*	MK575453	—	SDR NAMA 2017-303	USA	—	—
*C. amethystina*	*C. zollingeri*	OM473852	—	iNaturalist 56881430	USA	—	—
*C. lilacina*	*C. zollingeri*	MK427064	—	ZP-1267 (MHHNU 7767)	China	—	—
*C. lilacina*	*C. zollingeri*	MW374244	—	rxsbn-523	China	—	—
*C. lilacina*	*C. orientalis*	PQ819512	PQ814271	MHHNU6801	China	—	—
*C. lilacina*	*C. orientalis*	PQ819513	PQ814272	MHHNU7352	China	—	—
*C. lilacina*	*C. orientalis*	PQ819514	PQ814273	MHHNU7586	China	—	—
*C. lilacina*	*C. orientalis*	PQ819515	PQ814274	MHHNU7767	China	—	—
*C. lilacina*	*C. orientalis*	PQ819516	PQ814275	MHHNU32116	China	—	—
*C. lilacina*	*C. zollingeri*	OR567605	—	JAC14886 (PDD 107102)	New Zealand	—	—
*C. lilacina*	*C. zollingeri*	OR567670	—	JAC17787 (PDD 114897)	New Zealand	—	—
*C. lilacina*	*C. zollingeri*	OR567562	OR567721	JAC13837 (PDD 106042)	New Zealand	—	—
*C. lilacina*	*C. zollingeri*	**PV399840**	**PV399849**	PDD 81263	New Zealand	5.7 × 4.6	1.26
*C. lilacina*	*C. zollingeri*	**PV399841**	**PV399850**	PDD 94782	New Zealand	5.8 × 4.8	1.22
*C. lilacina*	*C.* aff. *zollingeri*	KP257143	KP257212	PBM3386 (TENN070808)	Australia	—	—
*C. tongdaoensis*	*C. tongdaoensis*	PQ819517	PQ814276	MHHNU11091	China	—	—
*C. tongdaoensis*	*C. tongdaoensis*	PQ819518	PQ814277	MHHNU11093	China	—	—
*C. tongdaoensis*	*C. tongdaoensis*	PQ819519	PQ814278	MHHNU11094	China	—	—
*C. violaceopulchra*	*C. zollingeri*	**PV603503**	—	BRACR29005	Czech Republic	5.6 × 4.9	1.13
*C. violaceopulchra*	*C. zollingeri*	**PV399842**	**PV399850**	G00576171	France	6.2 × 5.2	1.18
*C. violaceopulchra*	*C. zollingeri*	**PV603504**	—	O-F-175336	Norway	6.1 × 5.2	1.17
*C. violaceopulchra*	*C. zollingeri*	**PV603511**	**—**	BRACR15130	Slovakia	6.1 × 4.8	1.26
*C. violaceopulchra*	*C. zollingeri*	**PV603505**	—	BRACR23959	Slovakia	6.1 × 4.8	1.28
*C. violaceopulchra*	*C. zollingeri*	**PV603506**	—	BRACR30724	Slovakia	5.9 × 4.6	1.28
*C. violaceopulchra*	*C. zollingeri*	**PV603507**	—	BRACR30732	Slovakia	6.1 × 4.8	1.27
*C. violaceopulchra*	*C. zollingeri*	**PV603510**	—	BRACR41672	Slovakia	5.9 × 5.2	1.14
*C. violaceopulchra*	*C. zollingeri*	**PV603508**	—	BRACR30735	Slovakia	—	—
*C. violaceopulchra*	*C. zollingeri*	**PV603509**		BRACR15909	Slovakia	—	—
*C. violaceopulchra*	*C. zollingeri*	**PV399843**	**PV399852**	ARAN-Fungi 22716	Spain	6 **×** 4.8	1.25
*C. violaceopulchra*	*C. zollingeri*	**PV399844**	**PV399853**	BIO-Fungi 12557	Spain	5.7 **×** 4.8	1.18
*C. violaceopulchra*	*C. zollingeri*	**PV399845**	**PV399854**	ARAN-Fungi A3078104	Spain	6 × 5.2	1.16
*C. violaceopulchra*	*C. zollingeri*	OP538791	—	2.1.424	Switzerland	—	—
*C. violaceopulchra*	*C. zollingeri*	OP538785	—	2.1.35	Switzerland	—	—
*C. violaceopulchra*	*C. zollingeri*	OP538705	—	14_100a	Switzerland	—	—
*C. violaceopulchra*	*C.* sp.	OR858736	—	iNaturalist 162964275	USA	—	—
	*C. fumosa*	KP257126	KP257199	TFB11839 (TENN060724)	Russia	—	—
	*C.* cf. *fumosa*	KP257127	HQ877697	TENN043695	USA	—	—
	*C.* cf. *rubicundula*	HQ877696	JN214482	MR00170 (TENN064094)	USA	—	—

## Data Availability

Publicly available datasets were analyzed in this study. These data can be found here: https://www.ncbi.nlm.nih.gov (accessed on 9 April 2025).
